# VNIR-SWIR Hyperspectral Fusion-Based Multi-Task Detection Method: A Case Study on Fruit Origin-Category Authentication and Bruise Detection

**DOI:** 10.3390/foods15132381

**Published:** 2026-07-03

**Authors:** Bing Li, Chaofan Huang, Wei Tao, Shan Zeng, Chaoxian Liu, Yixiao Wang, Zhiguang Yang

**Affiliations:** School of Mathematics and Computer Science, Wuhan Polytechnic University, Wuhan 430023, China

**Keywords:** hyperspectral imaging, multi-task learning, hyperspectral fusion, quality inspection, origin-category authentication, bruise detection

## Abstract

Artificial intelligence-assisted food detection is increasingly moving from single-task classification toward integrated analytical systems capable of producing multiple quality-related outputs from one sensing workflow. However, most hyperspectral food detection studies still rely on a single spectral range or simple feature concatenation, which limits their ability to exploit complementary physicochemical information from heterogeneous sensors. In this study, an artificial intelligence-enabled visible–near-infrared and short-wave infrared (VNIR-SWIR) hyperspectral fusion framework is proposed for multi-task fruit detection, using origin authentication and bruise localization as representative tasks. The proposed method first constructs an observation-consistent fused representation from high-resolution VNIR images and low-resolution SWIR images. Collaborative spectral unmixing is used to couple cross-modal material distributions, while abundance-consistency and downsampled observation-consistency constraints are introduced to estimate SWIR-informed features on the VNIR spatial grid without assuming measured high-resolution SWIR ground truth. The fused representation is then processed by a shared spectral–spatial deep encoder with two task-specific heads: a fruit-level classification head for origin authentication and a pixel-level segmentation head for bruise detection. Experiments on apple and kiwifruit datasets show that the proposed framework outperforms VNIR-only, SWIR-only, bicubic-fusion, CNMF-style fusion, and TV-regularized fusion baselines under five fruit-level stratified random splits. For origin-category authentication, the proposed method achieved an accuracy of almost 93.85 for apples and almost 94.35 for kiwifruit. For bruise localization, the proposed method achieved higher overall accuracy, average accuracy, and Cohen’s kappa across the evaluated fruit categories.

## 1. Introduction

Artificial intelligence-assisted food detection is increasingly shifting from single-task quality classification toward integrated analytical systems capable of generating multiple quality-related outputs from one sensing workflow [1,2,3]. Compared with conventional laboratory-based analytical methods, hyperspectral imaging combined with machine learning or deep learning can provide rapid, nondestructive, and spatially resolved information on food quality, authenticity, and safety [1,2]. This capability is particularly important for postharvest fruit inspection, where several analytical decisions may be required for the same sample. For example, fruit origin authentication supports authenticity verification, regional-brand protection, and supply-chain quality control, whereas bruise detection is essential for reducing postharvest loss and preventing damaged products from entering commercial distribution [4,5,6,7]. These two tasks differ substantially in analytical scale and output form: origin authentication is usually performed at the fruit level based on global spectral fingerprints, while bruise detection requires pixel-level localization of weak and spatially heterogeneous tissue damage.

Hyperspectral imaging is attractive for these tasks because it integrates spectral and spatial information within one imaging modality [1,2]. Visible–near-infrared (VNIR) hyperspectral imaging generally provides high spatial sampling and captures pigment-related absorption, peel color, and surface-scattering characteristics, which may reflect differences in cultivar background, maturity, orchard environment, and postharvest handling [2,4]. Short-wave infrared (SWIR) hyperspectral imaging is more sensitive to water, carbohydrates, dry matter, and organic-matrix absorptions, which are closely related to internal tissue properties and bruise-induced changes [8,9,10]. Recent studies have shown that VNIR-SWIR information can improve the analysis of food components, fungal contamination, and quality-related chemical attributes, indicating the value of complementary spectral ranges for food analytical applications [8,9,10].

However, VNIR and SWIR sensors are not two directly interchangeable views of the same signal. They differ in spectral response, spatial resolution, penetration depth, signal-to-noise characteristics, illumination sensitivity, and acquisition geometry. Therefore, dual-sensor fusion is not simply a matter of increasing the number of wavelengths or stacking spectral bands. Previous studies using VNIR-SWIR fusion have demonstrated the potential of multi-source hyperspectral information for food and agricultural product inspection, but many fusion strategies still rely on low-level concatenation, feature-level combination, or decision-level integration [10,11]. For pixel-level bruise detection, small registration errors or SWIR upsampling artifacts may shift lesion boundaries, generate false local structures, or dilute weak bruise responses. This makes observation consistency particularly important when constructing fused hyperspectral representations from heterogeneous sensors.

The fusion problem is therefore deeper than increasing the number of wavelengths. For sample-level origin authentication, a small spatial misregistration may have a limited effect because the decision is based on global spectral statistics. For pixel-level bruise detection, however, even small registration errors or SWIR upsampling artifacts can shift lesion boundaries, create false local structures, or dilute weak bruise signals. Full-spectrum high-resolution acquisition over 400–2500 nm could reduce this problem, but such systems are costly and involve trade-offs among spectral coverage, spatial sampling, scanning speed, and signal-to-noise ratio.

Recent machine learning and deep learning studies have achieved promising results in food detection tasks such as origin identification, quality prediction, bruise detection, and foreign matter detection [2,3,5,6,7]. Nevertheless, many existing approaches still focus on a single spectral range, a single analytical task, or an independent task-specific model. Such designs may increase computational redundancy and limit their applicability in integrated inspection workflows. Multi-task learning provides a natural AI framework for this problem because a shared encoder can extract common spectral–spatial features while task-specific heads can generate heterogeneous outputs, such as fruit-level origin labels and pixel-level bruise maps [12]. Recent discussions on food structure, function, and artificial intelligence have further emphasized the need for integrated AI-based analytical systems in food-quality assessment [13]. Recent studies have also demonstrated the potential of hyperspectral imaging combined with multi-task learning for simultaneous detection or prediction of multiple food quality attributes [14,15].

Despite these advances, dual-sensor VNIR-SWIR fusion has not been sufficiently investigated as the foundation for AI-enabled multi-task fruit detection. Existing origin-category authentication studies mainly emphasize fruit-level classification performance based on global spectral or chemical fingerprints, whereas bruise-detection studies usually focus on local defect segmentation using one spectral range. The novelty of the present work lies in treating dual-sensor fusion as an observation-consistent representation-learning problem rather than a simple input concatenation problem. In particular, the reconstructed SWIR-informed component is constrained to remain consistent with the measured low-resolution SWIR observation after spatial degradation, which helps reduce the risk of generating visually sharpened but spectrally implausible features. Few previous food-inspection studies have explicitly connected this type of physically constrained VNIR-SWIR fusion with a shared multi-task framework that supports both sample-level and pixel-level outputs.

**Figure 1:**
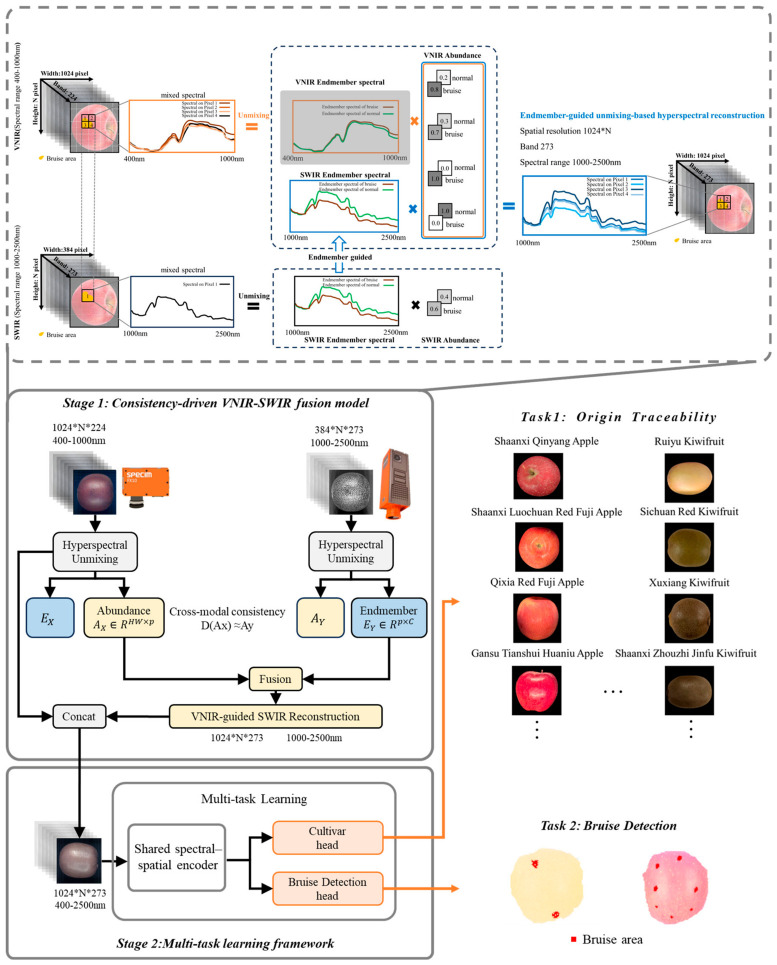
Schematic diagram of the proposed framework.

To address this issue, this study proposes an artificial intelligence-enabled VNIR-SWIR hyperspectral fusion framework for multi-task fruit detection, as shown in Figure 1. Origin-category authentication and bruise localization are selected as representative tasks because they require different levels of spectral–spatial information. The proposed method first uses collaborative spectral unmixing to connect cross-modal material distributions between VNIR and SWIR data. Abundance-consistency and downsampled observation-consistency constraints are introduced to estimate SWIR-informed features on the VNIR spatial grid while maintaining agreement with measured SWIR observations. The resulting fused representation is then processed by a shared spectral–spatial encoder with two task-specific heads for fruit-level origin-category authentication and pixel-level bruise detection.

(1) We formulate dual-sensor VNIR-SWIR hyperspectral fusion as an observation-consistent representation-learning problem for multi-task food inspection, rather than as simple band concatenation. The proposed workflow produces both origin-category labels and bruise probability maps from one aligned fused input, demonstrating the potential of hyperspectral fusion for heterogeneous nondestructive quality-assessment tasks.

(2) We introduce an observation-consistent VNIR-guided SWIR estimation strategy based on collaborative spectral unmixing. The method uses abundance consistency to encourage cross-modal structural correspondence and downsampled observation consistency to ensure agreement with measured SWIR data. The estimated SWIR-informed component is therefore interpreted as a physically constrained representation on the VNIR grid, not as directly measured high-resolution SWIR data.

(3) We evaluate the proposed fusion-driven multi-task framework through case studies on apple and kiwifruit origin-labeled categories and induced bruise samples. The validation strategy includes same-backbone input comparisons, input-level ablations, verified repeated random-split analysis, expanded bruise-segmentation metrics, and measured computational profiling.

The remainder of this paper is organized as follows. Section 2 describes the fruit materials, dual-sensor VNIR-SWIR imaging systems, preprocessing, consistency-driven fusion module, and multi-task learning network. Section 3 reports spectral analysis, origin-category-authentication results, bruise-detection results, and ablation analysis. Section 4 discusses the implications and limitations of the proposed framework and concludes the paper.

## 2. Materials and Methods

### 2.1. Sample Preparation

**Figure 2:**
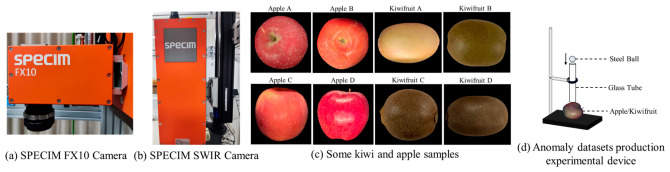
Hyperspectral imaging instruments and data collection process.

Apples and kiwifruits were used to construct a dual-sensor hyperspectral dataset for evaluating fusion-driven multi-task learning in fruit origin-category authentication and bruise detection. Four apple origin-labeled categories as shown in Figure 2 were included: Shaanxi Qinyang (Apple A), Shaanxi Luochuan Red Fuji (Apple B), Qixia Red Fuji (Apple C), and Gansu Tianshui Huaniu (Apple D). Four kiwifruit origin-labeled categories were included: Ruiyu (Kiwifruit A), Sichuan Red Heart (Kiwifruit B), Xuxiang (Kiwifruit C), and Shaanxi Zhouzhi Jinfu (Kiwifruit D). In this study, origin-category authentication is implemented as supervised classification among predefined origin-labeled categories. Fruits with visible mechanical damage, decay, or severe shape defects before bruise induction were excluded. A total of 800 samples were collected from fruits.

### 2.2. Task Definition

This study defines a dual-output setting to examine whether VNIR-SWIR hyperspectral fusion can support multi-task nondestructive fruit inspection. The first task is fruit-level origin-category authentication among predefined origin-labeled categories. Each fruit is assigned to one class according to its sampled provenance category. This task depends on global spectral–spatial fingerprints associated with pigment composition, peel scattering, maturity-related chemistry, cultivar background, and production-region effects. The second task is bruise detection, implemented as pixel-level binary mapping of bruised versus sound tissue. This task requires localized sensitivity to weak tissue damage and boundary-preserving spatial detail. VNIR information is expected to be useful for surface-related cues and fine spatial structure, whereas SWIR information may provide complementary absorption responses associated with water, carbohydrates, and tissue-matrix changes. The proposed framework, therefore, treats these two tasks as a controlled case study for assessing the broader potential of hyperspectral fusion in multi-task food inspection, while avoiding over-interpretation as full real-world traceability.

### 2.3. Bruise Induction and Annotation

Fruits were stored at 1.0 ± 0.5 °C and 90 ± 5% relative humidity to maintain quality before experiments. Imaging experiments were conducted at 25 ± 1 °C and 60 ± 5% relative humidity. Bruises were induced using a vertical guiding device with an inner diameter of 25.0 ± 0.1 mm and a length of 500 ± 1 mm. An AGCr15 steel ball was guided through the tube to impact the apple and kiwifruit surfaces at a controlled impact speed of approximately 3.13 m/s. The guiding tube reduced lateral deviation from the target impact position, and the same impact protocol was used across samples to obtain localized mechanical bruises for pixel-level detection. The time interval between bruise induction and hyperspectral imaging was 3 min, and this interval was kept consistent for all samples. Because biological variability can affect bruise size and visibility, lesion locations were marked after impact and used for subsequent annotation and evaluation [16].

Pixel-wise bruise annotations were produced on VNIR-resolution grids. A false-colour image generated from bruise-sensitive bands was used only as an annotation aid. All masks were manually delineated by two trained annotators using ENVI 6.2 software following the same annotation guidelines. The annotators independently delineated bruise boundaries and were blinded to the model predictions. Disagreements were resolved by consensus to obtain the final binary bruise masks. Background pixels were removed using a binary fruit mask obtained by Otsu thresholding on a VNIR intensity image, followed by morphological closing. The resulting masks were used as reference labels for the pixel-level bruise-detection task, and all segmentation metrics were calculated only within the fruit mask.

### 2.4. Data Acquisition

VNIR hyperspectral images were acquired from 400 to 1000 nm (224 bands) using a SPECIM FX10 camera which sourced from Specim, Spectral Imaging Ltd. (Oulu, Finland) with 1024 spatial samples and a pixel size of 8 × 8 μm. SWIR images were acquired from 1000 to 2500 nm (273 bands) using a SPECIM SWIR camera which sourced from Specim, Spectral Imaging Ltd. (Oulu, Finland) with 384 spatial samples and a pixel size of 24 × 24 μm. Two 50 W halogen lamps were positioned symmetrically at 300 mm from the sample surface to reduce specular reflections. The working distance from the camera to the sample was set to 300 mm for both cameras. Line-scan acquisition was used. Exposure times were set to 6 ms for VNIR and 8 ms for SWIR, and the conveyor/scan speed was set to 15 mm/s. These settings were selected to avoid saturation while maintaining sufficient SNRs across the usable bands. For each imaging session, dark current and white reference images were recorded for radiometric calibration. The specific parameters of the hyperspectral imaging instruments are shown in Table 1.

### 2.5. Radiometric Calibration and Cross-Modal Registration

All processing was implemented in Python (v3.11) on a workstation equipped with an NVIDIA RTX 4090 GPU (24 GB) sourced from NVIDIA Corporation (Santa Clara, CA, USA). To obtain reliable spectral data, original images require correction through dark background subtraction and white reference calibration [15], as presented in Equation (1):

(1) $$I_{hc}\left(i,j\right)=\frac{I\left(i,j\right)-D\left(j\right)}{W\left(i,j\right)-D\left(j\right)}$$

where $I_{hc}\left(i,j\right)$ denotes the corrected relative reflectance spectrum at a certain position, $\left(i,j\right)$ represents the raw spectral data, $W\left(i,j\right)$ corresponds to the white reference data, and $D\left(j\right)$ indicates the dark current data. The variables *i* and *j* represent the row and column pixel positions within the spatial dimensions of the image, respectively. The VNIR and SWIR cubes were then registered to a common spatial coordinate system and used as the input for the subsequent fusion and multi-task learning procedures. VNIR and SWIR cubes were first registered to a common spatial coordinate system. A VNIR reference image was formed by averaging bands within 700–900 nm, and an SWIR reference image was formed by averaging bands within 1100–1300 nm. An affine transform T(⋅) mapping SWIR to VNIR coordinates was estimated using Enhanced Correlation Coefficient optimisation. The SWIR cube was then warped to VNIR coordinates and resampled onto the VNIR grid using bicubic interpolation. The downsampling operator D(⋅) used later in the loss functions was implemented by area-based resizing from the VNIR size $\left(H,W\right)$ to SWIR size $\left(h,w\right)$, ensuring consistent forward and inverse mappings.

### 2.6. Consistency-Driven Fusion and SWIR Super-Resolution via Collaborative Unmixing

The purpose of the fusion module is to construct a task-usable full-spectrum representation from two heterogeneous sensors without assuming access to measured high-resolution SWIR ground truth. Let the calibrated VNIR cube and SWIR cube be unfolded into matrix forms. A collaborative unmixing formulation was used to connect cross-modal material distributions with modality-specific spectral signatures. This design is motivated by the observation that fruit tissues may share coarse spatial material structures across sensors, while their spectral responses differ between the VNIR and SWIR ranges. A linear mixing model was adopted as a regularised approximation:

(2) $$\hat{X}=A_{x}E_{x},\hat{Y}=A_{y}E_{y},$$

where $A_{x}\in R^{HW\times p}$ and $A_{y}\in R^{hw\times p}$ are abundance matrices, and $E_{x}\in R^{p\times c}$, $E_{y}\in R^{p\times C}$ are endmember matrices. The number of endmembers was set to p, which provided stable reconstruction while avoiding over-fragmentation of tissue components. $E_{y}$ was initialised by VCA-derived SWIR endmembers and then fine-tuned during optimisation.

To enforce cross-modal material consistency, abundance maps were constrained after scale matching:

(3) $$L_{ab}={∥D(A_{x})-A_{y}∥}_{F}^{2}.$$

The unmixing loss was defined as

(4) $$L_{unmix}=∥X-A_{x}E_{x}{∥}_{F}^{2}+∥Y-A_{y}E_{y}{∥}_{F}^{2}+\mu \;L_{ab},$$

With μ=1.0, a high-resolution SWIR estimate was then synthesised by combining VNIR-guided abundances with SWIR endmembers:

(5) $$Z^{\prime }=A_{x}E_{y}\in R^{HW\times C}.$$

A lightweight refinement network consisting of three residual blocks with 64 channels was applied to suppress artefacts. Because measured high-resolution SWIR ground truth was unavailable, the reconstruction was constrained by observation consistency: After degradation to the native SWIR grid, the estimated SWIR-informed representation should agree with the measured SWIR cube. A total-variation penalty was also used to reduce spurious spatial oscillations introduced by upsampling and cross-sensor registration uncertainty:

(6) $$L_{sr}=∥Y-D\left(\tilde{Z}\right){∥}_{F}^{2}+\nu \;L_{tv},\nu =10^{-4}.$$

Finally, the fusion-related loss is defined as $L_{\mathrm{fus}\;}=L_{\mathrm{unmix}\;}+L_{sr}$; moreover, the fusion-related loss was defined as a weighted combination of unmixing, abundance-consistency, observation-consistency, and regularisation terms. A virtual full-spectrum representation was then formed by concatenating the calibrated VNIR cube with the estimated SWIR-informed component on the VNIR grid. This representation is designed for downstream learning; it should not be interpreted as a directly measured high-resolution full-spectrum image.

(7) $$\overset{‾}{Z}=Concat\left(X,\tilde{Z}\right)\in R^{H\times W\times \left(c+C\right)}.$$

**Figure 3:**
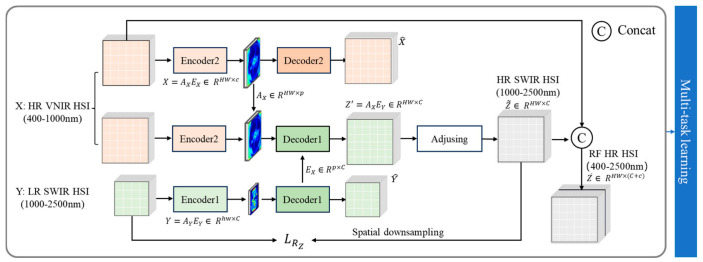
The architecture of the reconstruction module.

**Figure 4:**
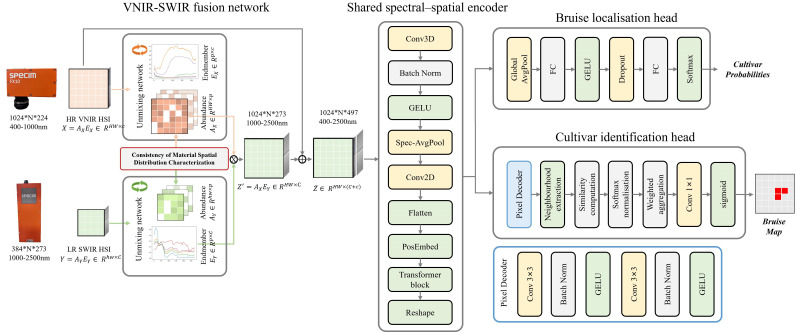
Schematic diagram of the hyperspectral fusion and multi-task learning network.

The resulting fused input provides a geometrically aligned representation for both tasks. For origin-category authentication, it supplies broader spectral coverage for global provenance-related fingerprints. For bruise detection, it introduces SWIR-informed absorption cues while retaining the VNIR spatial grid needed for pixel-level mapping. The architecture of the fusion module and the overall multi-task network are illustrated in Figure 3 and Figure 4.

### 2.7. Multi-Task Learning Method for Fusion-Driven Fruit Inspection

#### 2.7.1. Input Representation and Preprocessing

The multi-task network was fed with the fused VNIR-SWIR representation. Each band was standardised using the mean and standard deviation computed from the training set only to prevent information leakage. The fused representation was used because the two target tasks require complementary information: origin-category authentication benefits from global full-spectrum fingerprints, whereas bruise detection benefits from local spatial detail and SWIR-informed responses related to tissue damage. Feeding both tasks with one aligned representation also avoids inconsistent preprocessing between independent task-specific models.

#### 2.7.2. Shared Spectral–Spatial Encoder

A shared encoder was designed to support fruit origin-category authentication and bruise detection within one model. The encoder had to capture weak global provenance-related fingerprints while remaining sensitive to fine local anomalies. Origin cues may be spatially diffuse and influenced by pigment, peel scattering, and compositional differences, whereas early bruises are local, boundary-sensitive, and easily confounded by surface non-uniformity. A hybrid backbone was therefore adopted. Local spectral–spatial features were first extracted by a 3D convolutional pathway, and long-range dependencies and cross-band interactions were then modelled by Transformer blocks, allowing the two tasks to share a coherent fused feature space.

#### 2.7.3. Fruit-Level Origin-Category Authentication Head

Origin-category authentication was formulated as fruit-level classification over predefined origin-labelled categories. The head was designed to emphasise global category-related characteristics while reducing interference from local bruises. The shared feature map was aggregated by global average pooling over spatial dimensions, producing a 128-dimensional fruit-level embedding for each sample. This embedding summarised overall spectral–textural statistics potentially associated with regional production conditions, cultivar background, maturity, and postharvest handling. The embedding was then mapped to origin-category logits by a lightweight two-layer MLP with a 128-64-K configuration, where K is the number of origin-labelled categories. GELU activation and dropout were used to improve optimisation stability and reduce overfitting. Training was supervised by cross-entropy loss.

#### 2.7.4. Pixel-Level Bruise Detection Head

Bruise localisation was formulated as a pixel-level binary detection task. Spatial detail had to be preserved while computational cost remained low for practical inspection workflows. A shallow pixel decoder was applied to enhance local anomaly patterns from the shared feature map F. The decoder consisted of two 3 × 3 convolutions with 64 channels. BatchNorm and GELU were applied after each convolution. A single-channel logit map was then produced by a 1 × 1 convolution, and a sigmoid activation yielded a bruise probability map at the input resolution. Early bruises often show low contrast and fragmented responses; therefore, centre–neighbour self-similarity reweighting was introduced on the decoder features. A 15 × 15 window was used. Cosine similarity was computed between the centre-pixel feature and neighbouring features, and softmax-normalised weights were used to aggregate neighbourhood information. To address class imbalance, weighted binary cross-entropy was used, with the positive class weight set to 5.0.

#### 2.7.5. Joint Objective and Optimisation Details

Model optimisation followed a staged protocol. First, the fusion module was trained in an unsupervised manner using paired VNIR-SWIR data only so that abundance-consistent unmixing and VNIR-guided SWIR estimation could be learned without high-resolution SWIR ground truth. After convergence, the reconstructed full-spectrum cube was generated and used to train the multi-task network. Origin-category authentication was supervised by fruit-level labels, and bruise localization was supervised by pixel-wise masks. A unified objective was used, $L=\lambda_{fus}L_{fus}+\lambda_{cls}L_{cls}+\lambda_{det}L_{det}$, where $L_{fus}$ was defined in Section 2.6 and included abundance-consistency unmixing and self-supervised super-resolution consistency, $L_{cls}$ was the cross-entropy loss for origin-category authentication, and $L_{det}$ was the weighted binary cross-entropy loss for bruise localisation. The training objective combines fusion loss, cross-entropy loss for origin-category authentication, and weighted binary cross-entropy loss for bruise localisation. The loss weights were set to λ_fusion = 0.1, λ_cls = 1.0, and λ_seg = 1.0 for all reported results. Optimisation was conducted with AdamW. The initial learning rate was 1 × 10^−4^, and the weight decay was 1 × 10^−4^. A cosine learning-rate schedule was adopted to improve convergence. Training was run for up to 100 epochs. Early stopping was applied with a patience of 10 using validation loss as the primary monitoring criterion. Gradient clipping with an L2-norm threshold of 1.0 was applied to stabilise Transformer training.

### 2.8. Model Training, Evaluation Protocol, and Implementation Details

All pixels and patches extracted from the same fruit were kept within the same split to avoid data leakage. Model optimisation followed a staged procedure. First, the fusion module was trained in an unsupervised manner using paired VNIR–SWIR data only so that abundance-consistent unmixing and VNIR-guided SWIR estimation could be learned without high-resolution SWIR ground truth. After fusion training converged, the reconstructed full-spectrum cube was generated and used to train the multi-task network. Cultivar identification was supervised by fruit-level labels, and bruise localisation was supervised by pixel-wise masks. If end-to-end fine-tuning is used, it should be reported as a separate experimental setting with its own loss weights and validation criterion.

Baseline methods were implemented under identical data partitions and preprocessing. VNIR-only baselines were trained for both origin-category authentication and bruise detection using the same train/validation/test splits as the proposed method, and all hyperparameters were tuned exclusively on the validation set. Cultivar recognition performance was assessed using overall accuracy and macro-averaged F1 scores, and confusion matrices were reported to analyse inter-cultivar confusions. Bruise localisation performance was evaluated using overall accuracy, average accuracy, and Cohen’s kappa, following common practice in hyperspectral inspection studies. Because bruise pixels are a minority class, reporting bruise-class F1 or IoU in the final version would further strengthen the evaluation. All models were implemented in PyTorch (Python 3.11) and trained on an NVIDIA RTX 4090 GPU (24 GB).

## 3. Experimental Results and Discussions

### 3.1. Spectral Analysis

Origin-labelled category authentication and bruise detection are expected to rely on partially different hyperspectral ranges. Category authentication may be influenced by provenance-associated, cultivar-associated, maturity-associated, and batch-associated differences in pigment composition, peel scattering, and organic-matrix composition. Bruise detection is associated with moisture redistribution, cell rupture, and tissue-structure disruption, which can alter absorption and scattering responses in the near-infrared and SWIR domains. These task-dependent spectral requirements provide the rationale for evaluating VNIR-SWIR fusion as the sensing basis for multi-output inspection while avoiding over-interpretation of the current classes as pure geographic-origin effects or as complete real-world traceability.

To make this spectral rationale explicit, Table 2 summarises representative wavelength intervals reported in the literature as relevant to provenance/category authentication and bruise formation, together with their biochemical or structural attribution. The wavelength intervals provide mechanistic support for using complementary VNIR and SWIR observations.

### 3.2. Quality of Fusion-Driven Reconstruction

A critical requirement for using fused data in downstream learning is that the reconstructed high-resolution SWIR cube preserves the spectral fidelity of the measured SWIR data while inheriting the spatial structure from the high-resolution branch. The proposed module achieves this by collaborative spectral unmixing with an abundance-consistency constraint, followed by VNIR-guided super-resolution of SWIR features and band concatenation to form a virtual full-spectrum cube.

Fusion quality was assessed using the downsample-consistency principle adopted in the reconstruction loss. Specifically, the reconstructed high-resolution SWIR cube was spatially downsampled to the native SWIR grid and compared with the measured SWIR cube using RMSE and a spectral angle mapper (SAM):

$$RMSE=\sqrt{\frac{1}{N}\sum_{n=1}^{N}∥y_{n}-{\tilde{z}}_{n}^{↓}{∥}_{2}^{2}},SAM=\frac{1}{N}\sum_{n=1}^{N}arccos\left(\frac{y_{n}^{⊤}{\tilde{z}}_{n}^{↓}}{∥y_{n}{∥}_{2}∥{\tilde{z}}_{n}^{↓}{∥}_{2}}\right),$$

where the two spectra denote the measured SWIR observation and the downsampled reconstructed SWIR estimate at pixel i. Across all fruit categories, the VNIR-guided SWIR estimate achieved RMSE = 0.0217 ± 0.0043 and SAM = 3.42 ± 0.76 on the validation set. These metrics evaluate whether the reconstruction remains consistent with the observed SWIR measurements after downsampling. Qualitative observations, such as sharper defect boundaries and fewer block-like artefacts, should be interpreted together with RMSE/SAM because visual sharpness alone does not prove spectral fidelity. Therefore, the fused cube is described as a reconstruction-assisted concatenation rather than a simple raw concatenation of heterogeneous inputs.

### 3.3. Multi-Task Performance on Origin-Category Authentication and Bruise Detection

This section evaluates fruit-level origin-category authentication and pixel-level bruise detection using the fused VNIR-SWIR representation. The proposed method refers to the corresponding branches within the multi-task framework. The original comparison with KNN, SVM, and 1D-CNN is retained as a simple baseline reference, while controlled input-level comparisons are used to conservatively assess the contribution of VNIR-SWIR fusion and consistency-constrained reconstruction.

#### 3.3.1. Fruit Origin-Category Authentication

**Figure 5:**
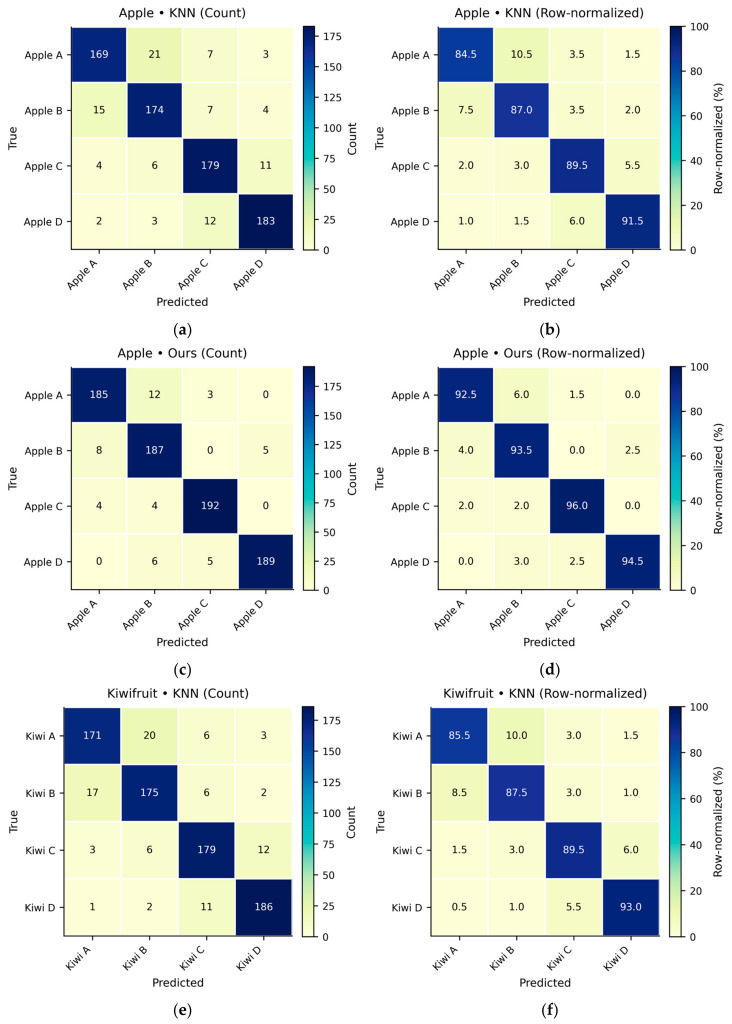

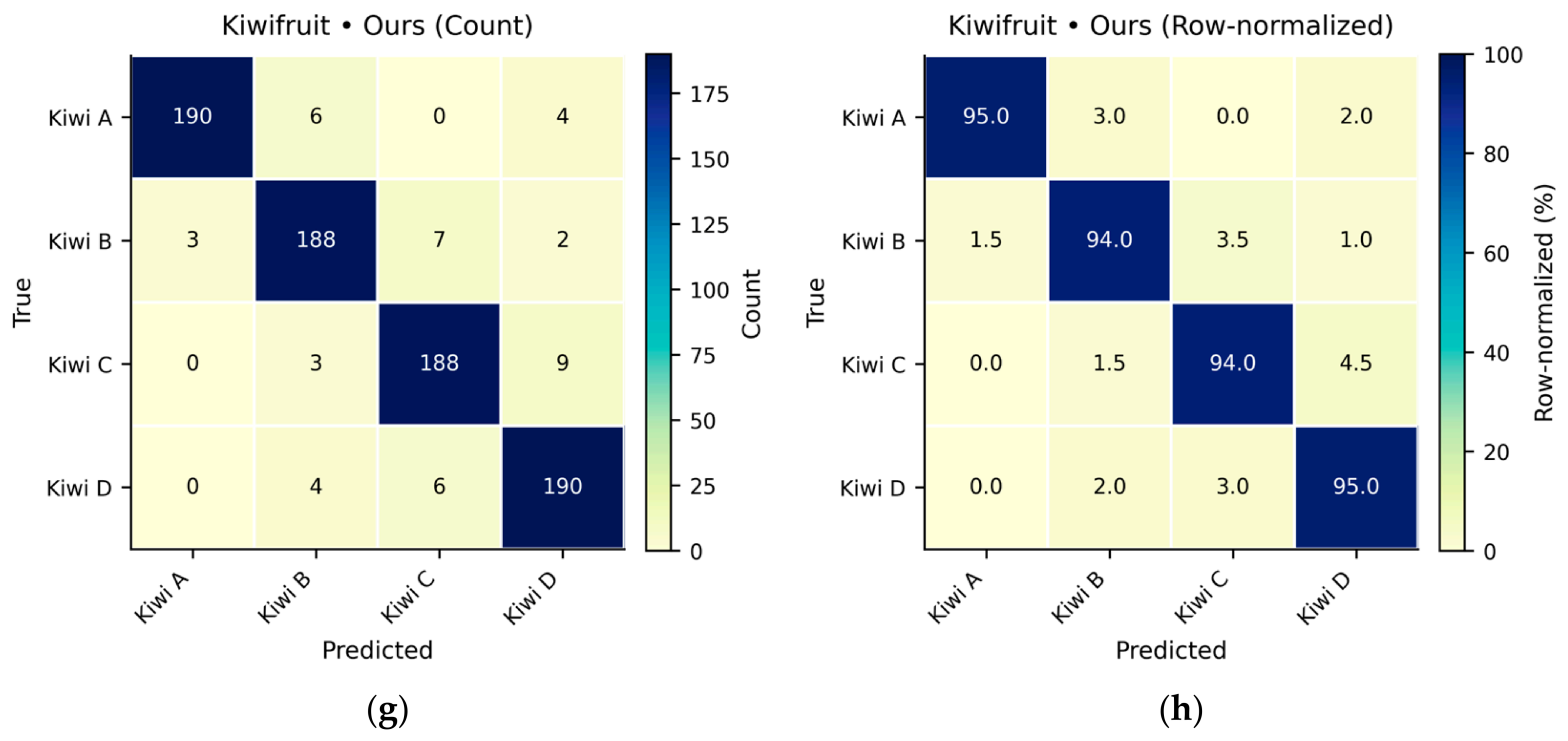
Confusion matrices for fruit origin-category authentication on apples and kiwifruits. (**a**) count results of KNN on apple; (**b**) row-normalized results of KNN on apple; (**c**) count results of ours method on apple; (**d**) row-normalized results of ours method on apple; (**e**) count results of KNN on kiwifruit; (**f**) row-normalized results of KNN on kiwifruit; (**g**) count results of ours method on kiwifruit; (**h**) row-normalized results of ours method on kiwifruit.

Origin-category authentication was evaluated under the fruit-level protocol described in Section 2.8. Overall accuracy (Acc) and macro-F1 are reported in Table 2, and class-wise precision, recall, and F1 are reported in Table 3. Confusion matrices are provided in Figure 5 to visualise the dominant inter-category errors. The reported values correspond to one fixed stratified train/validation/test split.

As shown in Table 3, the proposed multi-task model trained on the fused full-spectrum representation outperforms the VNIR-only KNN baseline for both fruit groups under the fixed-split evaluation. For apples, Acc increases from 88.13% to 94.13%, and macro-F1 increases from 88.12% to 94.15%. For kiwifruit, Acc improves from 88.88% to 94.50%, while macro-F1 improves from 88.86% to 94.52%. Because the test set is class-balanced, Acc is numerically aligned with the mean per-class recall. The simultaneous improvement in macro-F1 indicates improved origin-category authentication across classes rather than a gain confined to one category.

Table 4 shows that both precision and recall increase relative to KNN for all origin categories, leading to consistent F1 gains. The confusion matrices in Figure 5 show stronger diagonal dominance under the proposed method, while residual errors are concentrated among spectrally similar provenance categories within each fruit group. Nevertheless, because the baseline uses KNN whereas the proposed method uses a deep multi-task network, additional same-backbone ablations are needed to isolate the respective contributions of spectral fusion, model capacity, and multi-task learning.

Class-wise F1 gains range from 1.52 to 9.45 percentage points. For example, Apple A increases from 86.67% to 93.20%, and Kiwifruit A increases from 87.24% to 96.69%. Even for the category with the smallest improvement, which is Kiwifruit D, the F1 score increases from 92.31% to 93.83%. These results indicate consistent origin-category gains under the present dataset, but they should not be interpreted as generalised traceability beyond the tested origins, seasons, and sampling conditions.

To further characterise the remaining errors, confusion matrices are shown in Figure 5. Count matrices (left) and row-normalised matrices (right) are reported for the VNIR-only KNN baseline and the proposed multi-task model using the fused full-spectrum input. Panels (a–d) correspond to apples, and panels (e–h) correspond to kiwifruits. Row-normalised values represent per-class recall (%), highlighting the distribution of residual errors across provenance categories. Misclassifications are mainly concentrated among spectrally similar categories within each fruit group, while the proposed model strengthens diagonal dominance and reduces off-diagonal confusions.

#### 3.3.2. Bruise Detection

**Figure 6:**
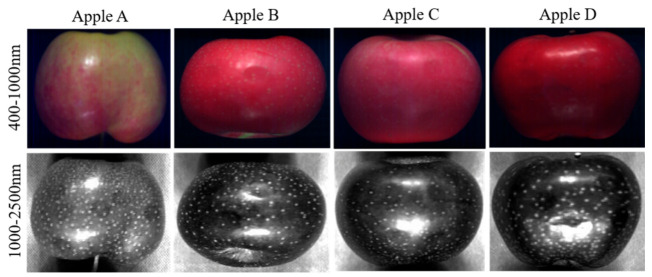
True-color and false-color images of apples.

The effectiveness of the proposed dual-source fusion framework for bruise localisation was evaluated by comparing SVM and 1D-CNN baselines trained on HR VNIR HSI with the proposed complete system using HR VNIR HSI and LR SWIR HSI. The comparison evaluates practical performance under the current baseline setting; however, same-input and same-backbone comparisons are required to isolate the effect of fusion from network architecture, decoder design, and model capacity. Hyperspectral data from different apple categories were used to validate bruise-area detection. In Figure 6, the first row displays HR VNIR HSI images within the 400–1000 nm spectral range as true-colour images, and the second row shows the corresponding LR SWIR HSI images covering 1000–2500 nm as false-colour images.

**Figure 7:**
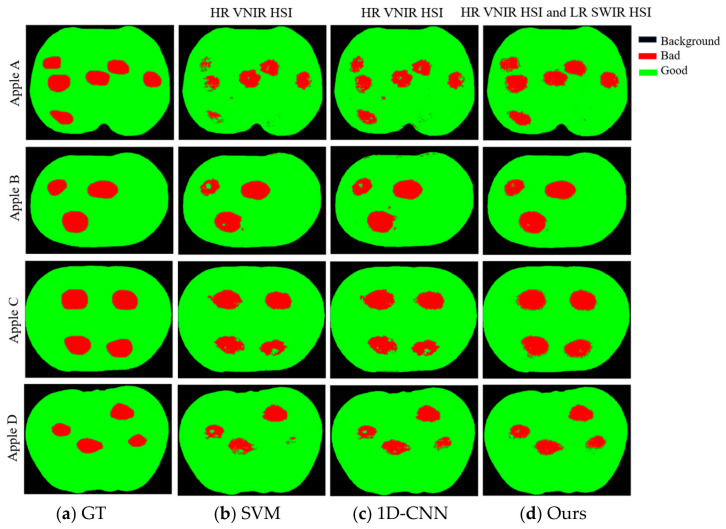
Detection results of different varieties of apple data using various bruise detection methods.

In Figure 6a–d, the side regions of Apple A, Apple B, Apple C, and Apple D exhibit six, three, four, and four bruise spots, respectively. Although the true-colour and false-colour images in Figure 6 faintly reveal the bruise areas, the boundaries of the bruise regions are difficult to discern. Figure 7 visualises bruise detection performance for the different apple cultivars. Figure 7a shows the ground-truth map, where green indicates healthy tissue, red marks bruised tissue, and black denotes background. Figure 7b,c present the outputs of SVM and 1D-CNN, respectively, using HR VNIR HSI as input. In the first row, both models miss the lesion centre and falsely identify several spurious lesion regions. Across rows 2–4, SVM performs poorly on lateral lesions. The 1D-CNN reduces some errors but still misclassifies healthy tissue as bruise and misses part of the lesion centre. Figure 7d shows that the proposed joint reconstruction and bruise-detection design produces more spatially coherent apple bruise maps under the tested conditions.

Table 5 further verifies the performance advantage of the proposed complete system in apple bruise detection under the selected metrics. Across all apple datasets, the proposed method attains higher OA, AA, and Cohen’s kappa than the SVM and 1D-CNN baselines. In the Shaanxi Qinyang lateral-bruise set, the proposed method achieves an AA of 96.3%, κ × 100 of 91.8, and OA of 98.3%. The AA surpasses those of SVM and 1D-CNN by 11.3 and 7.2 percentage points, respectively. Because κ accounts for chance agreement, it is more informative than OA when severe class imbalance produces high random concordance. However, OA, AA, and kappa are still insufficient for a complete pixel-level lesion evaluation; bruise-class precision, recall, F1, Dice, IoU, and AUPRC should be reported to demonstrate minority-class and boundary performance. The weaker performance of SVM and 1D-CNN is likely related to their limited ability to jointly exploit spatial context and SWIR-related information, although this interpretation must be confirmed using the same-input and same-backbone ablations.

**Figure 8:**
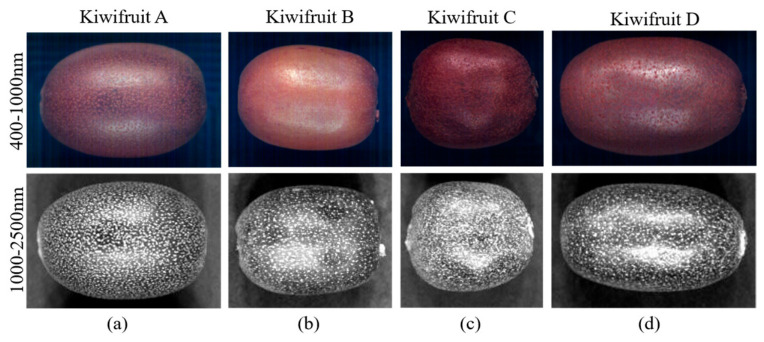
True-color and false-color images of kiwifruits.

**Figure 9:**
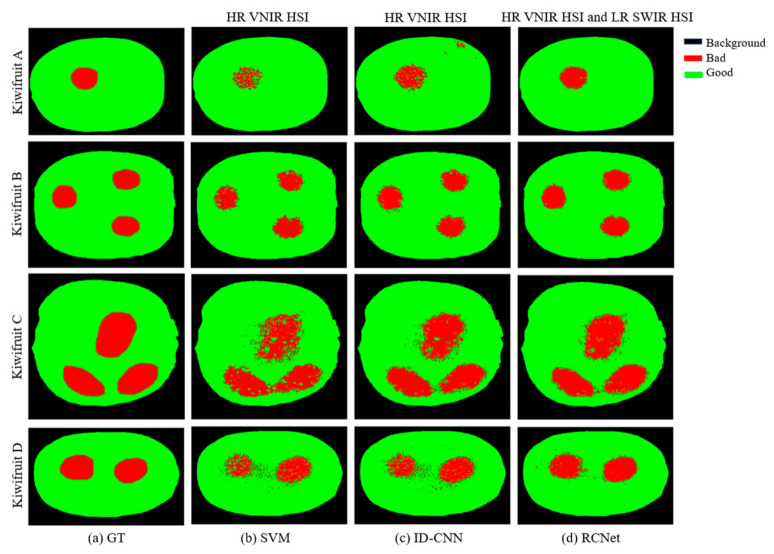
Detection results of different varieties of kiwifruit data using various bruise detection methods.

Hyperspectral images of bruised kiwifruit from four predefined categories are shown in Figure 8. The Ruiyu, Sichuan Red-Heart, Xuxiang, and Shaanxi Zhouzhi Jinfu samples contain one, three, three, and two lateral bruises, respectively. Figure 9 contrasts the bruise-detection maps produced by the competing methods. When only HR VNIR HSI is supplied, both the SVM and 1D-CNN misclassify two closely spaced lesions: the healthy tissue between them is erroneously labelled as a bruise, revealing weak discrimination near lesion boundaries and interiors.

The SVM further under-detects bruise pixels, whereas the 1D-CNN over-detects them and, in the Ruiyu sample, falsely marks peripheral healthy pixels as bruise area. By contrast, our method yields markedly cleaner maps. More true bruise pixels are recovered, virtually no peripheral healthy tissue is mislabelled, and only minor boundary shifts relative to the ground truth remain. Crucially, the model’s ability to resolve the narrow gap between adjacent lesions is substantially improved.

Table 5 further evaluates the classifiers on the kiwifruit bruise dataset. Averaged over the four kiwifruit cultivars, the proposed method achieves OA = 98.23%, AA = 96.48%, and κ × 100 = 92.50. These average values exceed those of SVM by 2.48, 7.55, and 14.68 percentage points, respectively, and exceed those of the HR-VNIR-only 1D-CNN by 1.53, 4.88, and 9.05 percentage points, respectively. For the Kiwifruit A subset, the proposed method obtains OA = 99.6%, AA = 97.9%, and κ × 100 = 95.1. These results indicate that the proposed framework improves early kiwifruit bruise localisation under the current dataset. The improvement is consistent with the use of complementary VNIR-SWIR information; however, because the current experiment compares input settings rather than isolated spectral bands, the band-level contribution of SWIR absorptions should be interpreted conservatively.

### 3.4. Ablation of the Dual-Source Fusion Setting

Removing any of the three fusion components reduces performance for both fruit groups, as shown in Table 6. The largest accuracy drop is observed when observation consistency is removed, indicating that constraining the reconstructed SWIR-informed representation to remain compatible with the measured SWIR observation after downsampling is important for robust dual-sensor fusion.

**Table 6:** Component-level ablation of the proposed fusion module.

Fruit Group	Variant	Origin Acc	Bruise F1/Dice
Apple	Full model	93.85 ± 0.75	93.41 ± 0.92
Apple	Without abundance consistency	92.67 ± 1.13	92.64 ± 0.85
Apple	Without observation consistency	91.42 ± 1.01	92.45 ± 0.82
Apple	Without refinement blocks	92.86 ± 0.81	92.89 ± 0.87
Kiwifruit	Full model	94.35 ± 0.65	94.26 ± 0.83
Kiwifruit	Without abundance consistency	92.93 ± 0.75	93.29 ± 0.71
Kiwifruit	Without observation consistency	92.37 ± 0.79	92.63 ± 0.93
Kiwifruit	Without refinement blocks	93.16 ± 0.60	93.46 ± 0.56

### 3.5. Statistical Robustness and Same-Backbone Input Comparison Analysis

To strengthen statistical robustness, the key same-backbone comparisons were evaluated using repeated fruit-level stratified random splits. Table 7 reports the controlled input comparison for origin-category authentication. Compared with the VNIR-only 3D-CNN + Transformer setting, the proposed observation-consistent VNIR-SWIR fusion improved apple accuracy from 90.52 ± 1.15% to 93.85 ± 0.75% and kiwifruit accuracy from 91.20 ± 1.25% to 94.35 ± 0.65%. The proposed method also showed higher mean accuracy than the bicubic VNIR-SWIR fusion comparator across the repeated splits.

For bruise localization, Table 8 expands the evaluation from OA, AA, and kappa to metrics that are more informative under severe class imbalance. Precision, recall, F1-score/Dice coefficient, IoU, and AUPRC were calculated within the fruit mask and averaged over repeated splits. The proposed method achieved F1/Dice values of 93.41 ± 0.92% for apples and 94.26 ± 0.83% for kiwifruit, outperforming both SVM and 1D-CNN baselines. Its AUPRC values reached 0.958 ± 0.008 for apples and 0.965 ± 0.006 for kiwifruit, indicating stronger robustness for minority bruise pixels.

### 3.6. Discussion

This study shows that observation-consistent VNIR-SWIR fusion can provide a more effective representation for multi-task fruit inspection than single-range imaging or simple interpolation-based fusion. The advantage of the proposed method lies not only in combining wider spectral information but also in constraining the reconstructed SWIR-informed component to remain consistent with the measured low-resolution SWIR observation. This is important because VNIR and SWIR sensors differ in spatial resolution, spectral response, penetration depth, and acquisition geometry. Without such constraints, fused data may contain visually enhanced but spectrally unreliable information.

The comparative results indicate that VNIR and SWIR information play complementary roles. VNIR data provide high spatial detail and surface-related cues, which are useful for boundary-sensitive bruise localization and appearance-related category differences. SWIR data provide additional absorption information associated with water, carbohydrates, dry matter, and tissue-matrix changes, which can support both category discrimination and bruise detection. Compared with bicubic fusion and representative hyperspectral fusion baselines, the proposed method better balances spatial enhancement and spectral consistency.

The single-task and multi-task comparisons suggest that the shared spectral–spatial encoder contributes to the final performance. Origin-category authentication depends mainly on global fruit-level spectral–spatial fingerprints, whereas bruise detection requires local pixel-level sensitivity to tissue damage. Although the two tasks differ in output form, they share relevant information related to pigment distribution, peel scattering, water status, and tissue structure. Therefore, the multi-task framework can learn common features while retaining task-specific prediction heads.

The component-level ablation further confirms the necessity of the main fusion components. Abundance consistency helps maintain cross-modal structural correspondence between VNIR and SWIR data. Observation consistency is particularly important because it prevents the reconstructed SWIR-informed representation from deviating from the measured SWIR observation. The refinement blocks help reduce spatial artifacts caused by registration, upsampling, and reconstruction uncertainty. These components jointly improve the reliability of the fused representation.

Several limitations should also be noted. First, the generated SWIR-informed component is a reconstructed representation rather than directly measured high-resolution SWIR data. Second, the current origin-category authentication task is based on predefined fruit categories and should not be over-interpreted as complete real-world traceability. Third, the bruises were induced under controlled experimental conditions, whereas natural bruises may vary in severity, depth, visibility, and time after impact. Future work should include broader multi-season and multi-origin validation, naturally bruised samples, stronger biochemical interpretation, and deployment-oriented model optimization for online food inspection.

## 4. Conclusions

This study proposed an observation-consistent VNIR-SWIR hyperspectral fusion framework for multi-task nondestructive fruit inspection, using origin-category authentication and bruise localization as representative tasks. The proposed method constructs a SWIR-informed representation on the VNIR spatial grid through collaborative spectral unmixing, abundance consistency, observation consistency, and lightweight refinement. This design avoids treating heterogeneous dual-sensor data as simple band concatenation and instead emphasizes physically constrained representation learning. The experimental results demonstrate that the proposed fusion-driven multi-task framework provides more effective spectral–spatial representations than single-range inputs, simple interpolation-based fusion, and representative hyperspectral fusion baselines. The single-task and multi-task comparisons further indicate that shared spectral–spatial encoding can provide consistent benefits for heterogeneous fruit-level and pixel-level detection tasks. Component-level ablation results confirm the importance of abundance consistency, observation consistency, and refinement blocks in constructing a reliable fused representation. Overall, this work shows the potential of consistency-constrained VNIR-SWIR hyperspectral fusion for integrated food-quality inspection. The framework provides a feasible route for generating both sample-level classification outputs and pixel-level defect maps from one unified sensing and learning workflow. Nevertheless, the present study remains a controlled case study based on selected fruit categories and induced bruising conditions. Future work should expand the validation to more seasons, orchards, cultivars, natural bruising scenarios, and online inspection environments.

## Figures and Tables

**Table 1:** The specific parameters of the hyperspectral imaging instrument.

Hyperspectral Imaging Instrument	SPECIM FX10	SPECIM SWIR
Spectral range	400–1000 nm	1000–2500 nm
Spectral bands	224	273
Spectral resolution	5.5 nm	10 nm
Spectral sampling/pixels	2.7 nm	5.6 nm
Spatial samples	1024	384
Pixel size	8 × 8 μm	24 × 24 μm
Maximum frame rate	330 FPS	450 FPS

**Table 2:** Representative wavelength intervals and mechanistic attribution for fruit origin-category authentication and bruise detection.

Wavelength Interval (nm)	Primary Attribution (Dominant Mechanism)	Relevance to Origin-Category Authentication	Relevance to Bruise Detection
400–550	Pigments (anthocyanins/carotenoids), epidermal colour	Strong: colour/pigment provenance cues	Indirect: may reveal surface discoloration in late bruises
650–750	Chlorophyll absorption/red-edge shift; scattering transition	Strong: red-edge/scatter differences	Moderate: damaged tissue may alter the red-edge slope
800–1000	NIR scattering, cell structure, water overtone shoulder	Moderate: structure and water status	Strong: water redistribution and structural disruption affect reflectance
~970	O–H overtone (free/weakly bound water)	Supplementary: water-status variation	Strong: moisture-related changes under bruising
1050–1200	C–H/O–H combinations and overtones; matrix composition	Moderate–strong: dry matter/matrix cues	Strong: bruise-related matrix changes often amplify differences
~1450	O–H first overtone (water)	Supplementary: hydration differences	Strong: sensitive to tissue hydration and bruise evolution
1900–2000	O–H combination band (water) and strong absorption	Limited: SNR-dependent water response	Often informative if SNR allows; reflects water status
2100–2350	C–H/C–O/N–H combinations (carbohydrates, organic matrix)	Moderate: carbohydrate/matrix cues	Moderate: tissue degradation can shift/reshape absorptions

**Table 3:** Fruit origin-category authentication performance.

Fruit Group	Input	Method	Overall Accuracy (%)	Macro-F1 (%)
Apple (4 origin-labeled categories)	HR VNIR HSI	KNN (baseline)	88.125	88.120
Apple (4 origin-labeled categories)	Fused VNIR-SWIR representation	Ours (multi-task)	94.125	94.145
Kiwifruit (4 origin-labeled categories)	HR VNIR HSI	KNN (baseline)	88.875	88.863
Kiwifruit (4 origin-labeled categories)	Fused VNIR-SWIR representation	Ours (multi-task)	94.500	94.515

**Table 4:** Class-wise fruit origin-category authentication performance.

Fruit Group	Method	Origin Category	Recall (%)	Precision (%)	F1 (%)
Apple	KNN (baseline)	Apple A	84.5	88.9	86.67
Apple	KNN (baseline)	Apple B	87.0	85.3	86.14
Apple	KNN (baseline)	Apple C	89.5	87.3	88.40
Apple	KNN (baseline)	Apple D	91.5	91.0	91.27
Apple	Ours (multi-task)	Apple A	92.5	93.9	93.20
Apple	Ours (multi-task)	Apple B	93.5	89.5	91.44
Apple	Ours (multi-task)	Apple C	96.0	96.0	96.00
Apple	Ours (multi-task)	Apple D	94.5	97.4	95.94
Kiwifruit	KNN (baseline)	Kiwi A	85.5	89.1	87.24
Kiwifruit	KNN (baseline)	Kiwi B	87.5	86.2	86.85
Kiwifruit	KNN (baseline)	Kiwi C	89.5	88.6	89.05
Kiwifruit	KNN (baseline)	Kiwi D	93.0	91.6	92.31
Kiwifruit	Ours (multi-task)	Kiwi A	95.0	98.4	96.69
Kiwifruit	Ours (multi-task)	Kiwi B	94.0	93.5	93.77
Kiwifruit	Ours (multi-task)	Kiwi C	94.0	93.5	93.77
Kiwifruit	Ours (multi-task)	Kiwi D	95.0	92.7	93.83

**Table 5:** Pixel-level bruise-detection evaluation results for apple and kiwifruit datasets.

Dataset		SVM (HR VNIR HSI)			1D-CNN (HR VNIR HSI)			Our Method (HR VNIR HSI and LR SWIR HSI)		
		OA (%)	AA (%)	K × 100	OA (%)	AA (%)	K × 100	OA (%)	AA (%)	K × 100
Apple	A	94.7	85.0	70.0	95.8	89.1	78.2	98.3	96.3	91.8
	B	98.6	96.2	92.5	98.7	97.5	93.0	99.3	98.2	96.2
	C	97.2	93.2	86.4	97.3	93.7	87.4	98.5	96.8	93.4
	D	97.3	89.4	78.7	98.6	95.0	89.9	99.0	95.9	92.7
Kiwifruit	A	98.0	86.4	72.8	98.3	90.1	81.2	99.6	97.9	95.1
	B	97.4	92.7	85.4	97.9	94.2	88.4	98.8	97.1	93.8
	C	92.9	89.2	78.3	95.2	92.9	85.8	96.5	95.1	90.0
	D	94.7	87.4	74.8	95.4	89.2	78.4	98.0	95.8	91.1

**Table 7:** Same-backbone comparison of different input and fusion settings.

Evaluation Setting	Method/Variant	Input/Fusion Setting	Backbone	Apple Acc (%)	Apple Macro-F1/F1-Dice (%)	Kiwifruit Acc (%)	Kiwifruit Macro-F1/F1-Dice (%)
Same-backbone comparison	Same backbone	VNIR only	3D-CNN + Transformer	90.52 ± 1.15	90.48 ± 1.12	91.20 ± 1.25	91.15 ± 1.28
Same-backbone comparison	Same backbone	SWIR only	3D-CNN + Transformer	85.60 ± 1.85	85.55 ± 1.82	86.45 ± 1.70	86.40 ± 1.74
Same-backbone comparison	Same backbone	Bicubic VNIR-SWIR	3D-CNN + Transformer	92.15 ± 0.95	92.10 ± 0.98	92.85 ± 0.90	92.80 ± 0.92
Same-backbone comparison	Ours/full model	Observation-consistent VNIR-SWIR	3D-CNN + Transformer	93.85 ± 0.75	93.88 ± 0.72	94.35 ± 0.65	94.38 ± 0.62
Same-backbone comparison	CNMF-style fusion	Coupled matrix factorization-based fusion	3D-CNN + Transformer	92.46 ± 0.84	92.73 ± 0.86	92.91 ± 0.79	93.21 ± 0.76
Same-backbone comparison	TV-regularized fusion	Variational/TV-regularized fusion	3D-CNN + Transformer	93.05 ± 0.80	93.02 ± 0.83	93.21 ± 0.77	93.52 ± 0.70

**Table 8:** Expanded bruise-segmentation metrics for repeated-split evaluation.

Fruit Group	Method	Input	Precision (%)	Recall (%)	F1/Dice (%)	IoU (%)	AUPRC
Apple	SVM	VNIR	76.43 ± 2.27	72.18 ± 2.91	74.12 ± 2.51	58.42 ± 3.35	0.715 ± 0.024
Apple	1D-CNN	VNIR	73.86 ± 2.62	84.47 ± 2.18	78.65 ± 2.24	64.38 ± 3.12	0.782 ± 0.018
Apple	Ours	Observation-consistent VNIR-SWIR	92.18 ± 1.07	94.82 ± 0.98	93.41 ± 0.92	87.21 ± 1.65	0.958 ± 0.008
Kiwifruit	SVM	VNIR	78.07 ± 2.19	75.34 ± 2.43	76.54 ± 2.31	61.55 ± 3.18	0.734 ± 0.020
Kiwifruit	1D-CNN	VNIR	75.28 ± 2.36	86.13 ± 1.91	80.18 ± 1.98	66.43 ± 2.85	0.805 ± 0.015
Kiwifruit	Ours	Observation-consistent VNIR-SWIR	93.47 ± 0.97	95.18 ± 0.83	94.26 ± 0.83	88.82 ± 1.45	0.965 ± 0.006

## Data Availability

The original contributions presented in this study are included in the article; further inquiries can be directed to the corresponding author.
